# Supplementary material for: Enhanced diagnosing patients suspected of sarcoidosis using a hybrid support vector regression model with bald eagle and chimp optimizers
Source: PeerJ Comput Sci. 2024 Dec 5;10:e2455. doi: 10.7717/peerj-cs.2455 (PMC11784889; doi:10.7717/peerj-cs.2455)
Supplement: Supplemental Information 2 [file peerj-cs-10-2455-s002.pdf]

Highlights of Data Preprocessing Steps

The data preprocessing steps in this study were designed to ensure the integrity and reliability of the dataset used for developing the predictive model. The key steps involved in the data preprocessing are as follows:

1. **Comprehensive Data Inspection**:
    - Conducted a thorough inspection of the dataset to identify any anomalies, inconsistencies, or potential issues that could affect data quality.
    - Confirmed the absence of missing data, ensuring the dataset's completeness and reliability.
2. **Outlier Detection**:
    - Utilized statistical methods, including z-scores and the interquartile range (IQR), to identify outliers.
    - Data points falling outside the acceptable range were flagged for further inspection.
3. **Outlier Imputation**:
    - Assessed identified outliers on a case-by-case basis.
    - Applied mean imputation to replace outliers with corrected values, ensuring they accurately reflected the underlying data distribution without introducing bias.
4. **Data Cleaning**:
    - Ensured the integrity and veracity of the dataset for subsequent analyses and modeling endeavors.

These preprocessing steps ensured a high-quality dataset, which is crucial for enhancing the accuracy and robustness of the predictive model for sIL-2R levels in sarcoidosis patients.
