# Supplementary material for: Enhanced diagnosing patients suspected of sarcoidosis using a hybrid support vector regression model with bald eagle and chimp optimizers
Source: PeerJ Comput Sci. 2024 Dec 5;10:e2455. doi: 10.7717/peerj-cs.2455 (PMC11784889; doi:10.7717/peerj-cs.2455)
Supplement: Supplemental Information 3 [file peerj-cs-10-2455-s003.pdf]

Description of Models Used

1. Support Vector Regression (SVR):

Support Vector Regression (SVR) is a powerful machine learning algorithm used for regression tasks. It aims to find an optimal hyperplane that accurately fits the training data within a certain tolerance margin. The key features of SVR include:

- **Kernel Trick**: SVR can handle non-linear data through kernel functions, such as radial basis function (RBF), polynomial, and linear kernels.
- **Regularization Parameter (C)**: Controls the trade-off between achieving a low training error and a low testing error, ensuring a smooth decision function.
- **Epsilon (ε)**: Defines a margin of tolerance where no penalty is given to errors, helping to make the model more robust.
- **Gamma (γ)**: A parameter for the RBF kernel that defines how far the influence of a single training example reaches, affecting the model's complexity.

2. Bald Eagle Search (BES):

The Bald Eagle Search (BES) algorithm is a nature-inspired optimization technique based on the hunting behavior of bald eagles. It involves three main stages:

- **Space Selecting**: Eagles select a promising area for prey based on their previous experiences and observations.
- **Space Searching**: Eagles search the selected area intensively to locate prey, updating their positions based on mathematical models.
- **Swooping**: Eagles swoop down to capture the prey, refining their positions to zero in on the optimal solution. The BES algorithm is used to optimize the hyperparameters of the SVR model, enhancing its predictive accuracy.

3. Chimp Optimizer (CO):

The Chimp Optimizer (CO) is another nature-inspired optimization algorithm modeled after the social and hunting behaviors of chimpanzees. It comprises three phases:

- **Group Formation**: Chimpanzees are divided into groups, each assigned a specific role based on their performance.
- **Hunting Phase**: Groups perform a local search, updating their positions guided by their roles and best-known positions.
- **Exploitation Phase**: Refinement of solutions found during the hunting phase, focusing on the most promising areas of the search space. The CO algorithm further refines the hyperparameters of the SVR model, improving its performance.

4. Hybrid BES-CO Optimization:

This novel hybrid approach combines the strengths of the BES and CO algorithms to optimize the hyperparameters of the SVR model. The process involves:

- **Initialization**: Setting up the initial population with positions and velocities for each agent.
- **BES Phase**: Performing global search, local search, and solution refinement to explore promising regions.
- **CO Phase**: Dividing agents into groups, performing a local search, and refining solutions. This hybrid optimization approach ensures a more precise and reliable prediction of sIL-2R levels, leveraging the complementary strengths of both BES and CO.

5. Firefly Algorithm (FFA):

The Firefly Algorithm (FFA) is an optimization algorithm inspired by the flashing behavior of fireflies. Key features include:

- **Attractiveness**: Fireflies are attracted to each other based on their brightness, which is associated with the objective function value.
- **Movement**: Fireflies move towards brighter ones, updating their positions to explore the search space. FFA is used to tune the hyperparameters of the SVR model, improving its convergence speed and accuracy.

6. Grey Wolf Optimization (GWO):

Grey Wolf Optimization (GWO) mimics the leadership hierarchy and hunting mechanism of grey wolves. It involves:

- **Encircling Prey**: Wolves update their positions based on the best positions found so far.
- **Hunting**: Wolves perform a local search, intensifying around the best-known positions.
- **Attacking Prey**: Wolves adjust their positions to converge on the optimal solution. GWO is used to adjust the hyperparameters of the SVR model, enhancing its performance.

7. Decision Tree (DT):

Decision Tree (DT) is a simple, yet powerful machine learning algorithm used for both classification and regression tasks. It works by recursively splitting the data into subsets based on the feature that provides the highest information gain or the lowest impurity. Key features include:

- **Interpretability**: Decision trees are easy to interpret and visualize.
- **Non-linearity**: They can capture non-linear relationships in the data.
- **Overfitting**: Prone to overfitting, which can be mitigated through pruning or ensemble methods.

8. Random Forest (RF):

Random Forest (RF) is an ensemble learning method that builds multiple decision trees and merges their results to improve predictive performance and control overfitting. Key features include:

- **Bagging**: Combines the predictions of multiple decision trees trained on different subsets of the data.
- **Feature Randomness**: Each split in a tree considers a random subset of features, enhancing diversity among trees.
- **Robustness**: Offers improved accuracy and robustness compared to individual decision trees.

The study evaluates the proposed hybrid SVR-BE-CO model against other optimization techniques, including SVR-FFA, SVR-GWO, and SVR-BES, as well as traditional machine learning models like Decision Tree (DT) and Random Forest (RF). These comparative analyses highlight the effectiveness of the hybrid model in enhancing predictive accuracy and robustness in clinical applications.
