# Supplementary material for: Enhanced diagnosing patients suspected of sarcoidosis using a hybrid support vector regression model with bald eagle and chimp optimizers
Source: PeerJ Comput Sci. 2024 Dec 5;10:e2455. doi: 10.7717/peerj-cs.2455 (PMC11784889; doi:10.7717/peerj-cs.2455)
Supplement: Supplemental Information 4 [file peerj-cs-10-2455-s004.pdf]

Study Motivation

Sarcoidosis is a complex, multi-system inflammatory disease primarily affecting the lungs and lymph nodes. Despite advancements in medical science, accurately predicting disease activity and response to treatment remains challenging. Traditional biomarkers, such as serum angiotensin-converting enzyme (ACE) levels, have shown limited reliability and inconsistent correlations with disease severity and progression. This inconsistency hampers the ability of clinicians to make informed decisions regarding patient management, leading to suboptimal treatment outcomes and unnecessary side effects from prolonged steroid usage.

The soluble interleukin-2 receptor (sIL-2R) has emerged as a promising biomarker for assessing disease activity in sarcoidosis. Elevated levels of sIL-2R correlate with T-cell activation and granuloma formation, key features of sarcoidosis. However, the clinical utility of sIL-2R remains underexplored, and its potential as a reliable indicator for predicting disease activity and treatment response warrants further investigation.

The recent advancements in machine learning offer a powerful toolset for predictive modeling in medical diagnostics. Support Vector Regression (SVR) is a robust machine learning technique known for its ability to handle non-linear data, making it suitable for complex medical predictions. However, the performance of SVR is highly dependent on the selection of its hyperparameters. Traditional optimization methods often fall short in efficiently navigating the large search space of hyperparameters, leading to suboptimal model performance.

In this study, we propose a novel hybrid optimization approach combining Bald Eagle Search (BES) and Chimp Optimizer (CO) to enhance the predictive accuracy of the SVR model for sIL-2R levels based on serum ACE levels. By leveraging the complementary strengths of BES and CO, our hybrid model aims to achieve a more precise and reliable prediction, thereby improving the clinical management of sarcoidosis.

Additionally, we compare the performance of our hybrid SVR model with other established optimization techniques, including the Firefly Algorithm (FFA), Grey Wolf Optimization (GWO), and BES alone. This comparative analysis provides insights into the effectiveness of different optimization strategies in enhancing the predictive power of SVR models.

The ultimate motivation behind this study is to develop a reliable, non-invasive predictive model that can aid clinicians in monitoring disease activity, tailoring treatments, and improving outcomes for patients suffering from sarcoidosis. By integrating advanced machine learning techniques with clinical data, we strive to contribute to the growing field of predictive modeling in healthcare and pave the way for personalized medicine approaches in managing complex diseases like sarcoidosis.
