# Supplementary material for: Enhanced diagnosing patients suspected of sarcoidosis using a hybrid support vector regression model with bald eagle and chimp optimizers
Source: PeerJ Comput Sci. 2024 Dec 5;10:e2455. doi: 10.7717/peerj-cs.2455 (PMC11784889; doi:10.7717/peerj-cs.2455)
Supplement: Supplemental Information 5 [file peerj-cs-10-2455-s005.pdf]

Limitations

While this study presents a novel approach to enhancing the predictive accuracy of sIL-2R levels in sarcoidosis patients using a hybrid SVR model with BES and CO optimizers, several limitations must be acknowledged:

1. **Dataset Size and Diversity**:
   - The dataset used in this study, while valuable, is limited in size and scope. It was obtained from a single medical center and covers a specific population. This may limit the generalizability of the findings to broader, more diverse populations. Future studies should validate the model on larger, more diverse datasets to ensure its applicability across different demographic and clinical settings.
2. **Hyperparameter Sensitivity**:
   - The performance of the SVR model is sensitive to the choice of hyperparameters. While the hybrid BES-CO optimization approach aims to find optimal hyperparameters, there is still a risk of overfitting or underfitting, especially when dealing with complex and high-dimensional data. Further refinement and validation of the hyperparameter tuning process are necessary to ensure robust model performance.
3. **Computational Complexity**:
   - The hybrid optimization approach combining BES and CO, while effective, is computationally intensive. This may limit its practicality in real-time clinical settings where quick decision-making is crucial. Streamlining the optimization process and exploring more computationally efficient algorithms could enhance the model's feasibility for clinical use.
4. **External Validation**:
   - The study primarily focuses on the development and internal validation of the predictive model. External validation using independent datasets from different clinical settings is essential to confirm the model's robustness and generalizability. Without such validation, the model's real-world applicability remains uncertain.
5. **Integration with Clinical Workflows**:
   - For the predictive model to be practically useful, it needs to be seamlessly integrated into existing clinical workflows. This includes ensuring compatibility with electronic health record systems and providing user-friendly interfaces for clinicians. The study does not address these integration challenges, which are critical for the successful implementation of the model in clinical practice.
